# THE DIRECT AND INDIRECT EFFECTS OF EDUCATION ON LATE-LIFE COGNITIVE ABILITY IN MEXICO

**DOI:** 10.1093/geroni/igz038.2902

**Published:** 2019-11-08

**Authors:** Joseph Saenz, Eileen M Crimmins, Elizabeth Zelinski

**Affiliations:** University of Southern California, Los Angeles, California, United States

## Abstract

Education and cognitive ability are closely associated. Less is known regarding mechanisms of this association. We evaluate direct effects of education on cognition and indirect effects through health and socioeconomic status (SES) in Mexico. We analyze adults age 50+ from the 2016 Mexican Cognitive Aging Ancillary Study (n=2,042). We constructed latent variables of visual and verbal cognitive abilities. Using structural equation modeling, we estimated direct effects of education on cognition and indirect effects through SES (income and wealth), and health (chronic conditions and health behaviors). Small, yet statistically significant, indirect effects of education on cognition through income, wealth, and stroke (for visual ability) and through stroke (for verbal ability) were observed. However, the majority of the association between education and cognitive ability (90% and 96% for visual/verbal cognitive ability, respectively) was not explained SES or health. Interventions to reduce disparities in late-life cognitive ability should address educational disparities in early-life.

